# Evaluation of Novel Quorum Sensing Inhibitors Targeting Auto-Inducer 2 (AI-2) for the Control of Avian Pathogenic Escherichia coli Infections in Chickens

**DOI:** 10.1128/spectrum.00286-22

**Published:** 2022-05-18

**Authors:** Yosra A. Helmy, Dipak Kathayat, Loic Deblais, Vishal Srivastava, Gary Closs, Robert J. Tokarski, Oluwatosin Ayinde, James R. Fuchs, Gireesh Rajashekara

**Affiliations:** a Center for Food Animal Health, Department of Animal Sciences, College of Food, Agricultural, and Environmental Sciences, The Ohio State University, Wooster, Ohio, USA; b Division of Medicinal Chemistry & Pharmacognosy, College of Pharmacy, The Ohio State University, Columbus, Ohio, USA; USDA-ARS

**Keywords:** APEC, quorum sensing, inhibitors, anti-virulence, auto-inducer 2, QSI-5, sulfadimethoxine, 5'-methylthioadenosine, chickens

## Abstract

Avian pathogenic Escherichia coli (APEC) associated with colibacillosis results in high morbidity and mortality, and severe economic losses to the poultry industry. APEC is a zoonotic pathogen and can infect humans through contaminated poultry products. Vaccination and antibiotic treatment are currently used to control APEC infections; however, the limited effect of vaccines and the emergence of antibiotic-resistant strains have necessitated the development of novel therapeutics. Here, we evaluated seven quorum sensing inhibitors (QSI) identified in our previous study, in APEC-infected chickens. QSIs were administered orally (~92 to 120 μg/bird) and chickens were challenged subcutaneously with APEC. Among them, QSI-5 conferred the best protection (100% reduction in mortality, 82% to 93% reduction in lesions [airsacculitis, perihepatitis, lung congestion, pericarditis] severity, and 5.2 to 6.1 logs reduction in APEC load). QSI-5 was further tested in chickens raised on built-up floor litter using an optimized dose (1 mg/L) in drinking water. QSI-5 reduced the mortality (88.4%), lesion severity (72.2%), and APEC load (2.8 logs) in chickens, which was better than the reduction observed with currently used antibiotic sulfadimethoxine (SDM; mortality 35.9%; lesion severity up to 36.9%; and APEC load up to 2.4 logs). QSI-5 was detected in chicken's blood after 0.5 h with no residues in muscle, liver, and kidney. QSI-5 increased the body weight gain with no effect on the feed conversion ratio and cecal microbiota of the chickens. Metabolomic studies revealed reduced levels of 5′-methylthioadenosine in QSI-5-treated chicken serum. In conclusion, QSI-5 displayed promising effects in chickens and thus, represents a novel anti-APEC therapeutic.

**IMPORTANCE** Avian pathogenic Escherichia coli (APEC), a subgroup of ExPEC, is a zoonotic pathogen with public health importance. Quorum sensing is a mechanism that regulates virulence, biofilm formation, and pathogenesis in bacteria. Here, we identified a novel quorum sensing autoinducer-2 inhibitor, QSI-5, which showed higher anti-APEC efficacy in chickens compared to the currently used antibiotic, sulfadimethoxine at a much lower dose (up to 4,500 times). QSI-5 is readily absorbed with no residues in the tissues. QSI-5 also increased the chicken’s body weight gain and did not impact the cecal microbiota composition. Overall, QSI-5 represents a promising lead compound for developing novel anti-virulence therapies with significant implications for treating APEC infections in chickens as well as other ExPEC associated infections in humans. Further identification of its target(s) and understanding the mechanism of action of QSI-5 in APEC will add to the future novel drug development efforts that can overcome the antimicrobial resistance problem.

## INTRODUCTION

Avian pathogenic Escherichia coli (APEC), is one of the major poultry pathogens causing significant economic losses to the poultry industry worldwide (1). APEC is an extra-intestinal pathogenic E. coli (ExPEC) and infections are characterized by a wide range of localized and systemic infections such as septicemia, salpingitis, airsacculitis, arthritis, peritonitis, yolk sac infection, swollen head syndrome, and respiratory tract infection (2, 3). APEC can be transmitted to humans through the consumption of contaminated poultry products and fresh produce that is amended with contaminated poultry manure (4). APEC strains share genetic similarity and virulence genes with human ExPECs such as uropathogenic E. coli (UPEC) and neonatal meningitis E. coli (NMEC) and has been reported to cause urinary tract infections and meningitis in rodent models and manifest in the form of foodborne gastrointestinal illnesses that are often accompanied by the ingestion of contaminated foods (4–6). APEC infects a wide range of poultry species of all ages and is common in chickens, turkeys, and ducks in different production systems and can negatively impact the body weight gain and the feed conversion ratio (2, 3).

APEC is prevalent in all ages of chickens, although, a higher prevalence is detected in adult layer chickens (36.7%) (7). At least 30% of the commercial poultry flocks in the United States have been estimated to be affected with colibacillosis at any given point of time (8). APEC infections also result in the reduction of meat production (2% reduction in chicken’s live weight), feed conversion ratio (up to 2.7%), and egg production (up to 15%), increased carcass condemnation at slaughter age (up to 45%) (5), and high morbidity and mortality of chickens (up to 20%) especially in young chickens (53.5% of the total mortality), leading to severe economic losses. Annual losses to the poultry industry in the United States due to APEC infection have previously been estimated at $40 million (9). Thus, APEC poses a significant threat to global poultry production as well as sustainable animal agriculture worldwide (5).

APEC infections in poultry are treated with antibiotics worldwide (quinolones, tetracycline, cephalosporins, aminoglycosides, sulfonamides, and colistin) and/or by vaccination (Poulvac E. coli). However, antibiotics have limited effect due to the emergence of multidrug-resistant (MDR) APEC strains and vaccine failure is associated with infection by heterologous serotypes (1, 10–12). For example, in the United States, Europe, and Australia, approximately 92% of APEC isolates with resistance to three or more classes of antibiotics, including to some of the most commonly used drugs such as tetracycline, streptomycin, and sulfonamides have been isolated (1). Furthermore, APEC isolates with resistance to colistin (possessing *mcr-1*) and β-lactam antibiotics (possessing extended-spectrum-β-lactamase, ESBL genotype) have been isolated from chickens in China, Egypt, and France (13, 14). Therefore, novel approaches are needed to effectively control APEC infections in poultry. Additionally, APEC is considered as a zoonotic foodborne pathogen and shares several important traits with human ExPEC (8). Thus, the control of APEC infections in poultry also has a public health significance.

**FIG 1:**
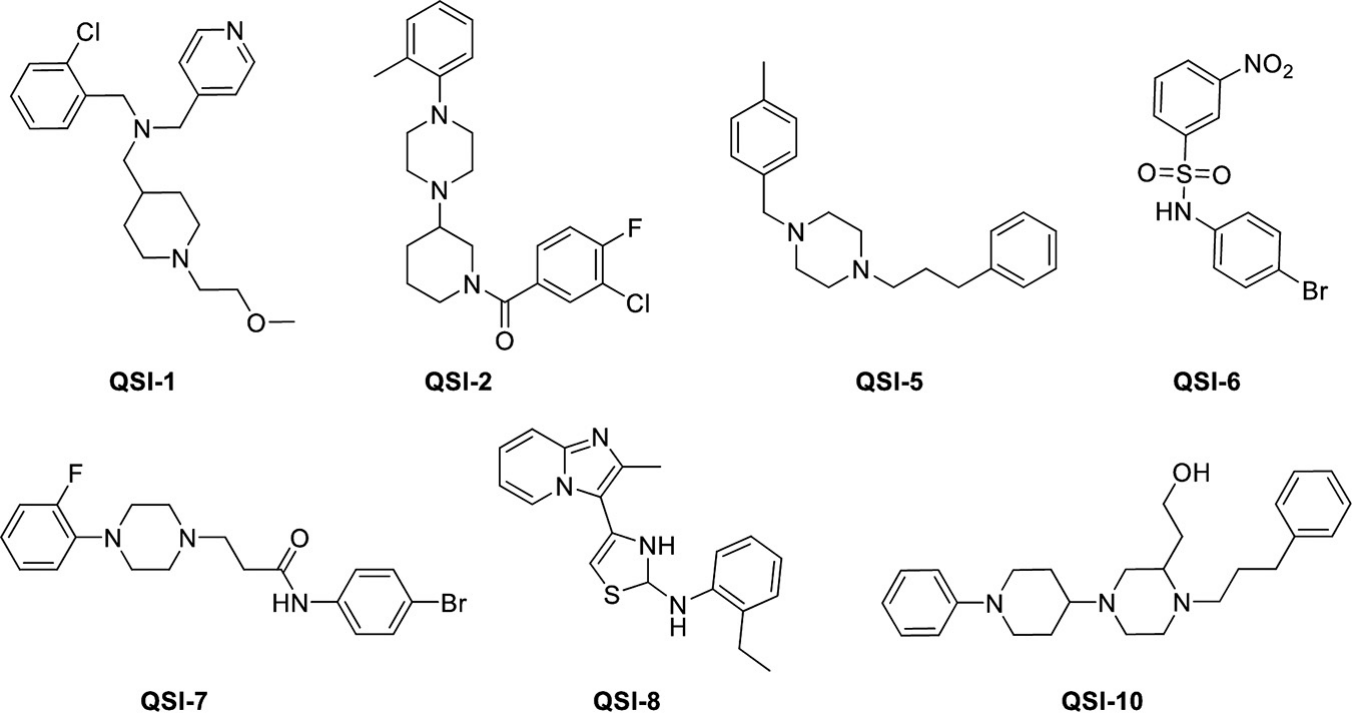
Chemical structures of the Quorum-Sensing AI-2 Inhibitors (QSIs) investigated in this study.

In several bacteria including E. coli, quorum sensing autoinducer-2 (QS AI-2) plays a critical role in virulence and biofilm formation (15). AI-2 is unique because it serves as the universal signal between interspecies QS communication for both Gram negative and Gram positive bacteria (16). In our previous study (17), we identified 10 novel QS AI-2 inhibitors by using Vibrio harveyi AI-2 indicator bacteria, that did not inhibit the APEC’s growth, but impacted the QS- regulated processes and showed efficacy against APEC infections *in vitro*. Previously, these quorum sensing inhibitors (QSI) were designated C-1 to C-10 (17); however, in the current study they are referred as QSI-1 to QSI-10. Here, we evaluated the efficacy of the seven (QS-1, -2, -5, -6, -7, -8, and -10; Fig. 1) potent AI-2 inhibitors from our previous *in vitro* studies in APEC-infected chickens. We then selected the best anti-APEC compound (QSI-5) that significantly reduced the mortality, APEC load and pathological lesion severity in infected chickens and (i) optimized its dose for delivery in drinking water; (ii) compared its efficacy with the currently used antibiotic (sulfadimethoxine, SDM) in a field simulated setting; (iii) evaluated its effect on gut microbiota and serum metabolites; (iv) measured its residue in muscle, liver, and kidney; and (v) conducted pharmacokinetic studies.

## RESULTS

### The QSI-5 and QSI-10 showed higher anti-APEC efficacy compared with other QSIs in chickens.

**FIG 2:**
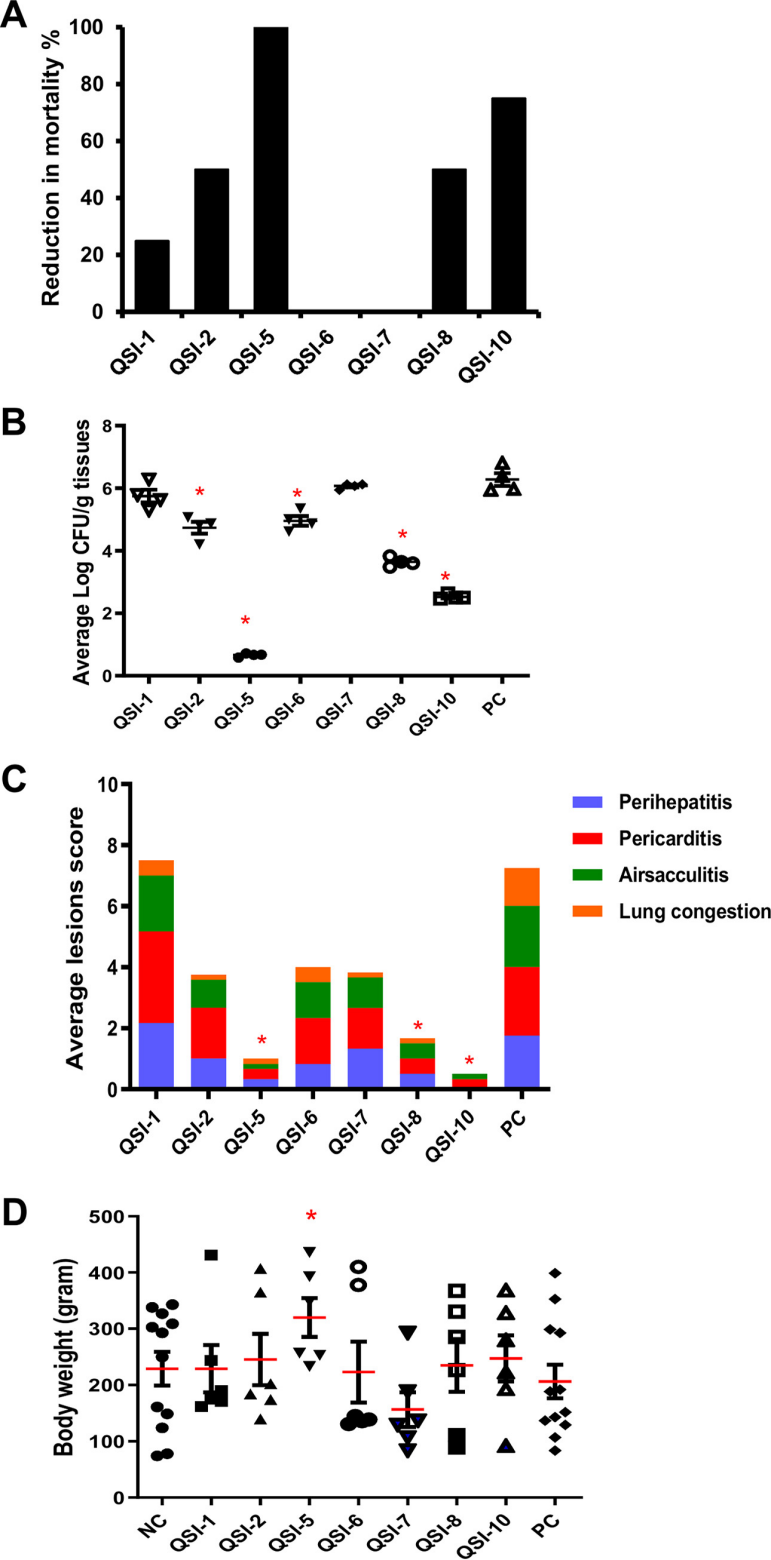
Effect of QSIs on (A) chicken's mortality, (B) APEC lesion severity in the internal organs (liver, heart, airsacs, and lung), (C) APEC load in the internal organs (liver, lung, heart, and kidney), and (D) body weight gain of QSIs-treated groups compared to the PC group. QSIs were administered once daily for 5 days using oral gavage and chickens were infected with APEC using s/c route. The average lesion score and APEC load was calculated, and the data were presented as an average of all the organs in each chicken and cumulative lesion scores for each group, respectively. *Significant difference between treated chickens (*P* < 0.05) and the PC group.

Treatment of 1-day-old chickens once daily with QSIs orally reduced the mortality, APEC load, and lesion severity in the internal organs compared to the positive control (PC; not treated and infected) group. To calculate the mortality reduction in the treated groups compared with the PC group, the mortality in the PC group was normalized to 100%. Our results showed that the treatment of chickens with QSI-5 and QSI-10 resulted in 100% and 75% reduction in mortality, respectively; while QSI-2 and QSI-8 resulted in a 50% reduction in mortality compared with the PC group. Further, treatment of chickens with QSI-1 reduced the mortality by 25%, while QSI-6 and QSI-7 did not reduce mortality compared to the PC group (Fig. 2A). Mortality details in QSIs-treated and control groups are shown in Table S1 and Fig. S1A.

The average APEC load reduction in the internal organs (liver, heart, lung, kidney) of QSI-5-treated chickens ranged between 5.2 and 6.1 logs CFU/g of tissues (*P* < 0.05); while the average reduction in APEC load in QSI-10 and QSI-8-treated chickens ranged between (3.4 to 4.3 logs; *P* < 0.05) and (2.2 to 3.1 logs CFU/g of tissues), respectively, compared with the PC group. Further, QSI-2 and QSI-6 reduced the APEC load between 1.1 logs and 1.8 logs CFU/g of tissues; whereas QSI-1 and QSI-7 did not reduce the APEC load compared with the PC group (Fig. 2B). Similarly, to calculate the reduction of lesion (perihepatitis, pericarditis, lung congestion, and airsacculitis) severity in internal organs, the lesion severity in the PC group was considered as 100%. The reduction of lesion severity in the QSI-5-treated group ranged between 78% and 93%, while QSI-10 resulted in 85% to 100% reduction of lesion severity compared to the PC group. Further, treatment of the chickens with QSI-8 resulted in 67% to 89% reduction of lesion severity, while QSI-2, QSI-6, QSI-7, and QSI-1 resulted in (26% to 89%), (33% to 67%), (11% to 89%), and (0% to 67%) reduction, respectively, compared with the PC group (Fig. 2C). None of the tested QSIs affected the body weight of treated chickens, except for QSI-5-treated chickens which showed increased average body weight (mean difference = 90.9 g) compared with negative control (NC; not treated and not infected) group (*P* < 0.05; Fig. 2D). Details of average APEC load and average lesion severity in each organ in QSIs treated and control groups are shown in Table S1.

### QSI-5 reduced the level of 5′-Methylthioadenosine, a component of QS AI-2 activated methyl cycle.

Untargeted metabolomic profiling was performed using LC-MS to determine the effect of QSIs (QSI-5, QSI-8, and QSI-10) treatment on chicken serum metabolites compared with the PC group. The QSI-5 treatment significantly reduced the level of 5′-methylthioadenosine (7 folds; *P *= 0.0009) that is involved in methionine metabolism pathway and spermidine and spermine biosynthesis pathway compared to the PC group (Table 1). Similarly, QSI-8 treatment significantly increased the abundance of all-trans-Carophyll yellow, a member of the triterpenoid family, (51.8 folds; *P* = 0. 000002); 9,10-DiHODE, a member of the linoleic acids, which are involved in lipid transport, lipid and fatty acid metabolism (3.6 folds; *P *= 0. 00008); and LysoPE (0:0/20:3[11Z,14Z,17Z]), a phospholipid, which is involved in the glycerophospholipid metabolism and lipid metabolism pathways (5.1 folds; *P *= 0. 0008), compared with the PC group. Treatment of chickens with QSI-10 increased the level of Tetranor-PGF1alpha, a prostaglandin (9.3 folds; *P* = 0. 0008) and LysoPE (20:4 (8Z,11Z,14Z,17Z)/0:0) (5.4 folds; *P* = 0. 0008), which are involved in glycerophospholipid metabolism and lipid metabolism pathways; while reduced the level of Betavulgaroside VIII, a member of the diterpene glycosides (17.6 folds; *P* = 0. 0002), which is involved in lipid metabolism pathway compared with the PC group (Table 1).

**TABLE 1:** The altered metabolites in chicken’s serum after the treatment with QSIs

QSI	Metabolites	Fold change^*a*^	*P*-value
QSI-5	5′-Methylthioadenosine	↓7.0	0.0009
QSI-8	all-trans-Carophyll yellow	↑51.8	0.000002
	LysoPE (0:0/20:3(11Z,14Z,17Z))	↑5.1	0.0008
	9,10-DiHODE	↑3.6	0.0008
QSI-10	Betavulgaroside VIII	↓17.6	0.0002
	Tetranor-PGF1alpha	↑9.3	0.0008
	LysoPE (20:4(8Z,11Z,14Z,17Z)/0:0)	↑5.4	0.0008

a Fold change was calculated by comparing to the abundance of metabolite in serum of the PC group. The arrows represent whether given metabolites are up (increased) or down (decreased) regulated.

To explain the variance of the metabolite profiles between treated and control groups, PCA was conducted with two principal components for QSI-5 (PC3 = 6.78%, PC5 = 3.5%), and QSI-8, and QSI-10 (PC1 = 30.5%, PC7 = 3.97%). The principal-component analysis (PCA) scores revealed that the metabolite profiles and composition of QSI-5-treated chickens clustered differently than the metabolites profile of PC (*P* = 0.04) and NC groups (0.0007) (Fig. S2A). Similarly, the metabolite profiles of QSI-8- (*P* = 0.0002) and QSI-10-treated (*P* = 0.009) chickens also clustered differently than the metabolite profiles of the PC group. Interestingly, metabolites profiles of QSI-8- and QSI-10-treated chickens clustered together (Fig. S2B).

### QSI-5 has no/minimal impact on the gut microbiota, QSI-10 significantly increased *Lactobacillus*, and QSI-8 significantly increased *Butyricicoccus* abundance.

**FIG 3:**
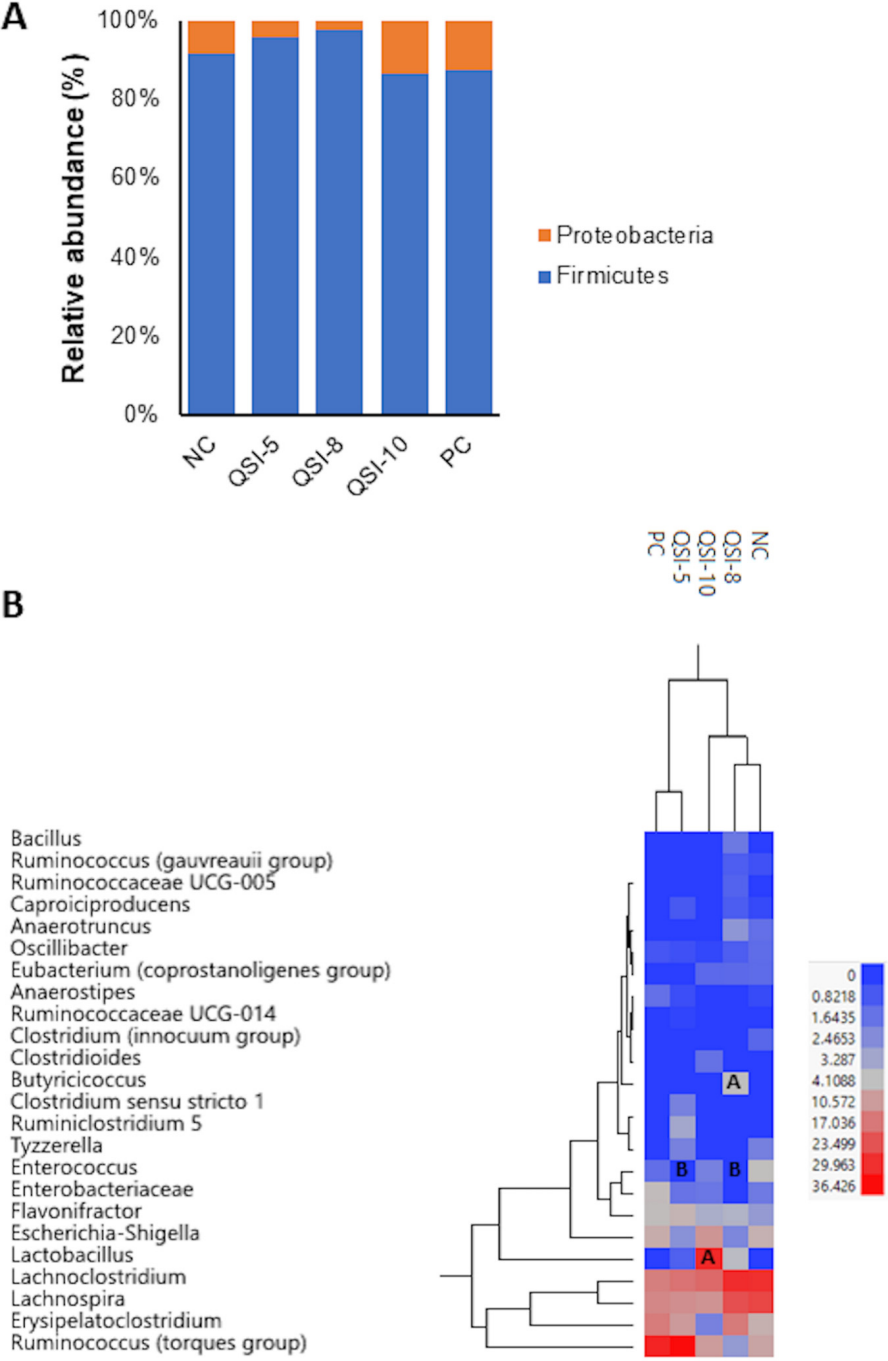
Relative abundance of gut microbial community at (A) phylum and (B) genus levels in QSI-treated chickens compared with the NC and PC groups. *Significant difference between treated chickens (*P* < 0.05) and the control groups. The heat maps generated using JMP PRO 13 software (SAS Institute). The letters A and B on the heat map indicate whether the OTUs were significantly increased or decreased, respectively, compared with the PC group (*P* < 0.05).

The alpha diversity analysis showed no difference in the phylogenetic diversity (*P* = 0.3; H = 5.1), richness (*P* = 0.1; H = 7.4), and evenness (*P* = 0.22; H = 5.7) of the gut microbiota between treated (QSI-5, QSI-8, and QSI-10) groups compared with both NC and PC (*P* <0.05) (Fig. S3A). Further, there was no spatial separation observed in the cecal microbiota of the QSIs-treated groups compared to the PC and NC groups when the principal coordinates analysis (PCoA) was performed using the unweighted uniFrac data (*P* <0.05) (Fig. S3B). Firmicutes was the most abundant bacterial phylum present in all chicken groups (87.3% to 95.7%) followed by Proteobacteria (4.3% to 13.7%). Interestingly, the treatment of chickens with QSI-5, QSI-8 and QSI-10 did not cause significant alterations in the gut microbiota compared to the NC or PC group at the phylum level (*P* > 0.05). Only QSI-5 and QSI-8 increased the Firmicutes (91.4% to 95%); whereas QSI-10 increased the Proteobacteria abundance (8.6% to 13.7%) compared with the NC group; however, this increase was not significant (Fig. 3A). Treatment of chickens with QSI-5 increased the abundance of *Ruminococcus* (torques group) (9% to 6%), *Flavonifractor* (2.9% to 6%), *Lactobacillus* (0% to 1.1%), *Clostridium sensu stricto* 1 (0% to 2.1%), *Ruminiclostridium* 5 (0%to 3.3%), and *Erysipelatoclostridium* (7.2% to 10.8%); while reduced *Enterococcus* (4% to 0%; *P* <0.05) compared with the NC group. Treatment of chickens with QSI-8 increased the abundance of *Butyricicoccus* (0% to 4.3%; *P* > 0.05), *Bacillus* (0% to 1.8%), *Lactobacillus* (0% to 4%), Erysipelatoclostridium (7.2% to 16.6%); while reduced *Enterococcus* (4.3% to 0%; *P* > 0.05) compared with the NC group. The high abundance of all the aforementioned genera explained the high abundance of phylum Firmicutes in the cecum of the QSI-5- and QSI-8-treated groups (Fig. 3B). Notably, treatment of chickens with QSI-10 significantly increased *Lactobacillus* (0% to 31%) abundance compared with the NC and PC groups (*P* <0.05) which explained the high abundance of phylum Firmicutes, while the increased abundance of Escherichia*-Shigella* (6.7% to 11.9%) explained the high abundance of phylum Proteobacteria (Fig. 3B).

### The optimal therapeutic dose of QSI-5 for treatment of APEC-infected chickens is determined to be 1 mg/L.

**FIG 4:**
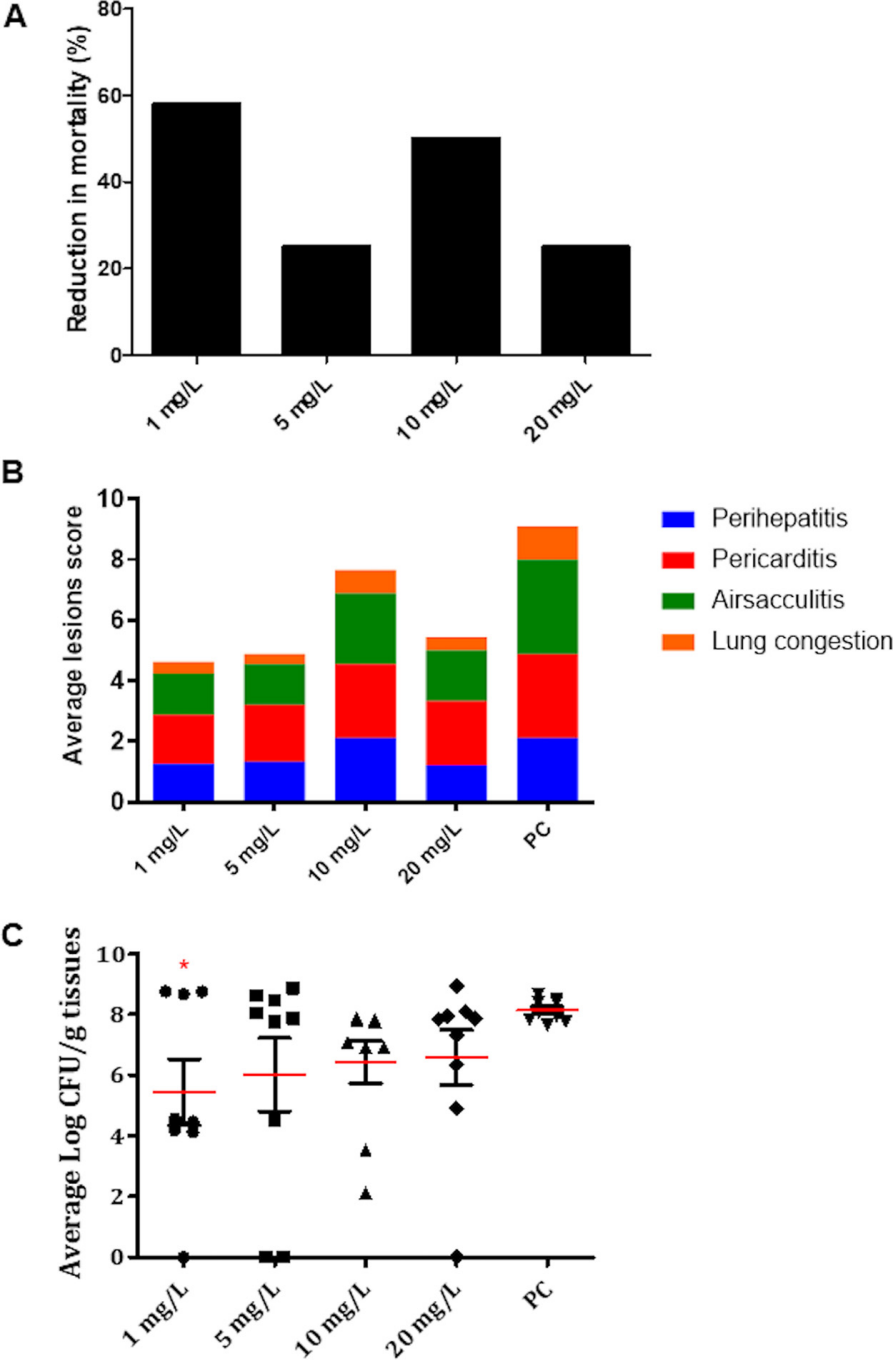
Effect of QSI-5 at 1 mg/L, 5 mg/L, 10 mg/L, and 20 mg/L on (A) chicken's mortality, (B) APEC lesion severity in the internal organs (liver, heart, airsacs, and lung), and (C) APEC load in the internal organs (liver, lung, heart, and kidney). QSI-5 was administered continuously in drinking water for 7 days and chickens were infected with APEC using s/c route. The average lesion score and APEC load was calculated, and the data were presented as an average of all the organs in each chicken and cumulative lesions score for each group, respectively. *Significant difference between treated and the PC group (*P* < 0.05) groups.

We optimized the dose of QSI-5 for drinking water delivery in chickens, a common industry practice, given that QSI-5 showed the best anti-APEC activity in chickens when administered by oral gavage in the pilot experiment above. Our results showed that treatment of chickens with 1 mg/L of QSI-5 reduced the mortality by 58%; while treatment with 5 mg/L, 10 mg/L, and 20 mg/L reduced the mortality by 25%, 50%, and 25%, respectively, compared with the PC group. The mortality observed in the PC group (60%) was normalized to 100% (Fig. 4A). Mortality details in QSI-treated and control groups are shown in Table S2 and Fig. S1B. Additionally, the reduction in pathological lesions (perihepatitis, pericarditis, airsacculitis, and lung congestion) in the 1 mg/L treated group ranged between 40.8% to 70%, which was higher than the reduction observed in the 5 mg/L (32% to 66.3%), 10 mg/L (0% to 30%), and 20 mg/L (24% to 60%) groups compared with the PC group (Fig. 4B). Similarly, APEC load in the internal organs (liver, heart, lung, kidney) of the 1 mg/L treated group was significantly (*P *< 0.05) reduced by approximately 2.3 to 3.1 logs CFU/g of tissues compared to the PC group; while the reduction ranged between (1.7 to 2.4), (1 to 2.6), and (1.3 to 1.9) logs CFU/g of tissues in 5 mg/L, 10 mg/L, and 20 mg/L treated groups, respectively, compared with PC group (Fig. 4C). Details about the average APEC load reduction and average lesion severity reduction in each organ in QSI-5-treated chickens at 1 mg/L, 5 mg/L, 10 mg/L, and 20 mg/L are shown in Table S2. The nature of this unexpected dose-response relationship is not clear but could conceivably be influenced by competing alternative mechanisms of action at higher concentrations.

### QSI-5 possessed higher anti-APEC efficacy than antibiotic SDM in chickens.

**FIG 5:**
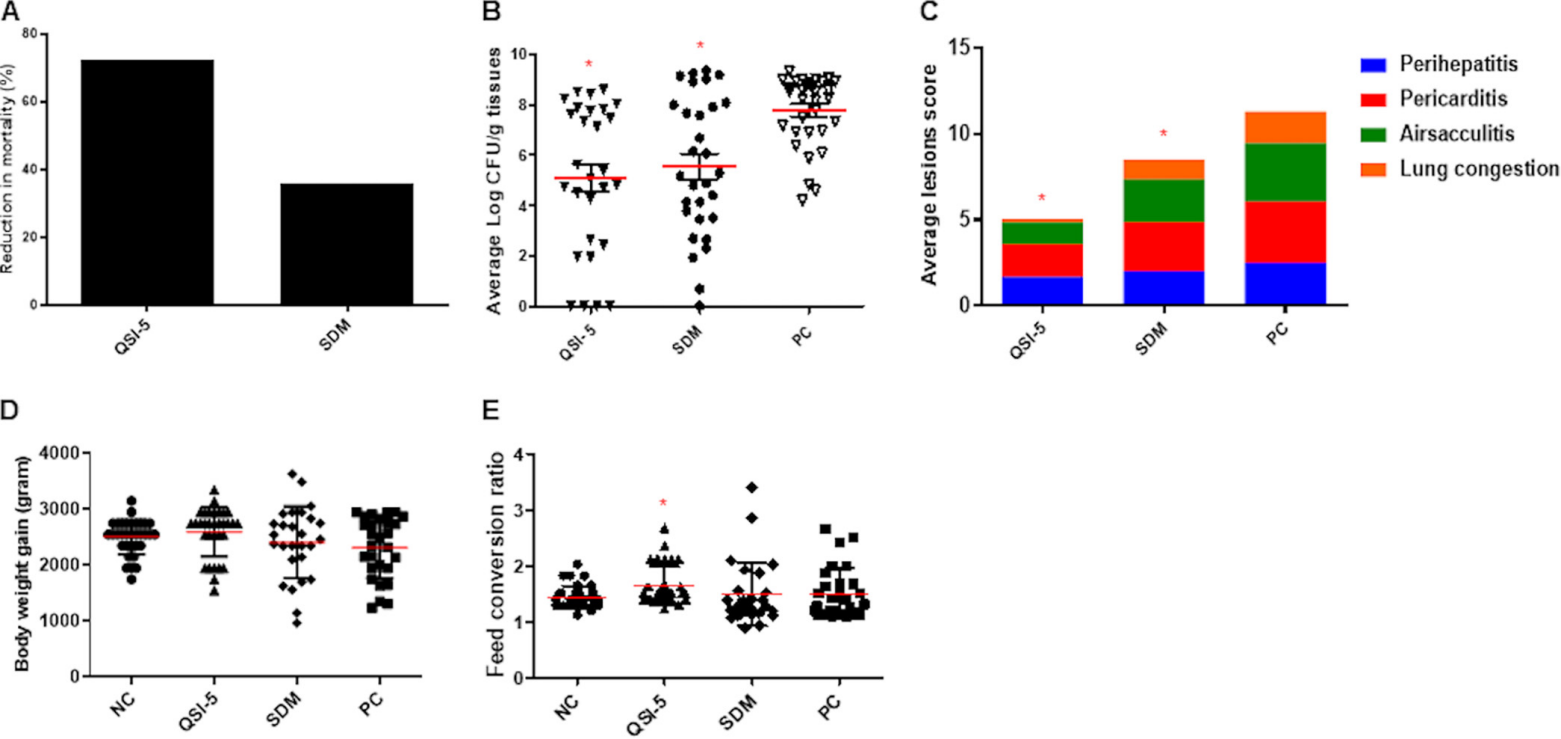
Comparing the efficacy of QSI-5 and sulfadimethoxine on (A) chicken’s mortality, (B) APEC lesion severity in the internal organs (liver, heart, airsacs, and lung), (C) APEC load in the internal organs (liver, lung, heart, and kidney), (D) body weight gain, and (E) feed conversion ratio. QSI-5 and sulfadimethoxine were administered continuously in drinking water for 7 days and chickens were infected with APEC using s/c route. The average lesion score and APEC load was calculated, and the data were presented as an average of all the organs in each chicken and cumulative lesions score for each group, respectively. *Significant difference between treated and control chicken (*P* < 0.05) groups.

We used the optimized dose of QSI-5 (1 mg/L) and therapeutic dose of SDM (0.05%/495.3 mg/L) to compare the efficacy of QSI-5 with SDM in commercial broiler chickens raised on built-up floor litter. Chickens treated with QSI-5 showed 72.2% reduction in mortality compared with the PC group; whereas a 35.9% reduction was observed in the SDM-treated group (Fig. 5A). Mortality details in the QSIs-treated and control groups are shown in Table S3 and Fig. S1C. In the QSI-5-treated group, APEC load in internal organs was reduced by 2.3 to 2.8 logs CFU/g of tissues compared with the PC group, depending on the organ; while the reduction was 1.9 to 2.4 logs CFU/g of tissue in SDM-treated group (Fig. 5B). Notably, on day 42, no APEC was recovered from any organs. Additionally, the reduction in APEC lesion severity in the QSI-5-treated group was 33.3% to 88.4%, which were higher than those observed in the SDM-treated group (from 19.2% to 36.9% reduction), respectively (Fig. 5C). Details about the average APEC load reduction and average lesion severity reduction in each organ of QSI-5- and SDM-treated groups are shown in Table S3. Further, body weight gain (BWG) and feed conversion ratio (FCR) were increased in the QSI-5-treated group (BWG: 2,659.1 g; FCR: 1.48) compared with the NC (BWG: 2,501.2 g; FCR: 1.46) and SDM-treated (BWG: 2,408.4 g; FCR: 1. 46) groups (Fig. 5D and E); however, this increase was not significant. Details about weekly BWG and FCR are shown in Table S4 and S5.

### QSI-5 was rapidly absorbed into chickens’ blood and no residues were detected in the chicken tissues.

**FIG 6:**
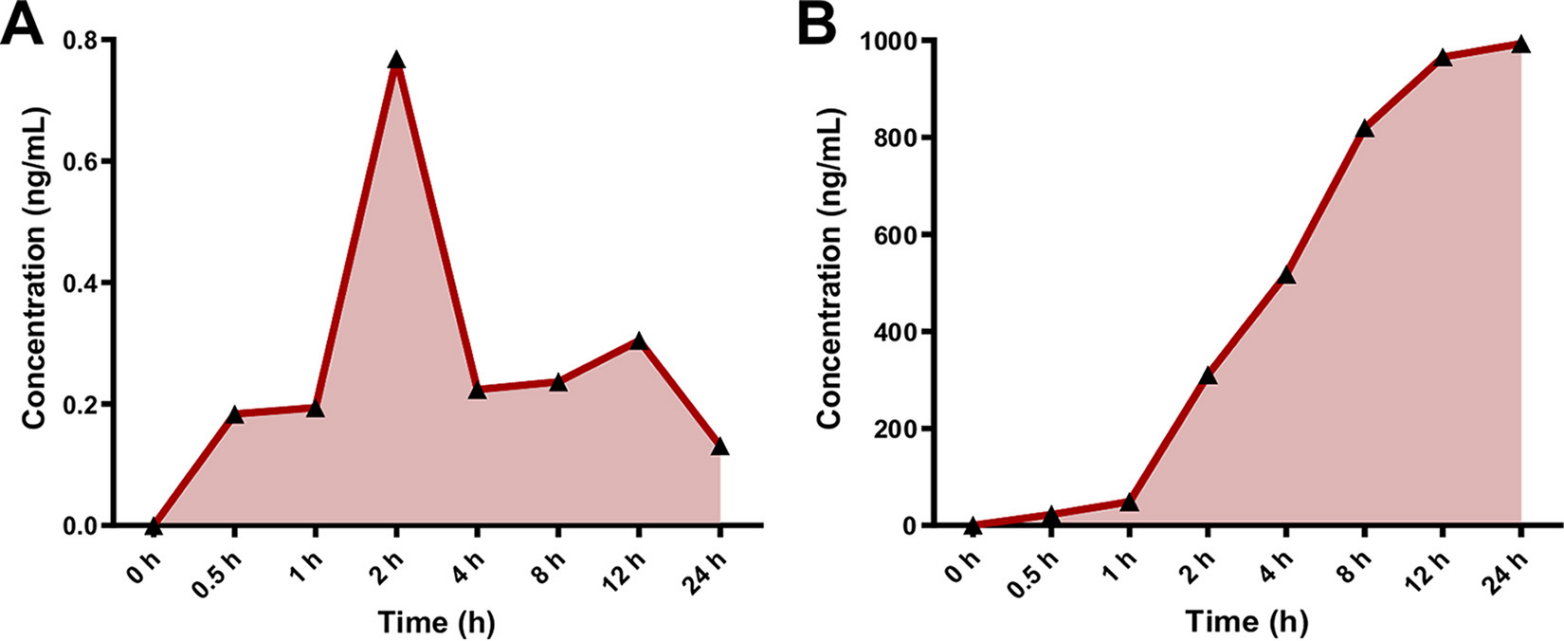
Pharmacokinetic profile of (A) QSI-5 and (B) sulfadimethoxine in chicken’s plasma. Data were analyzed using LC-MS. The PK profile was measured in five chickens per group at different time points (0 h, 0.5 h, 1 h, 2 h, 4 h, 8 h, 12 h, 24 h) after administration of the drugs.

The pharmacokinetic (PK) profile of QSI-5 in plasma and its residue in different tissues was analyzed using liquid chromatography-mass spectrometry (LC-MS). The PK profile was measured (five chickens/group) at each time point (0 h, 0.5 h, 1 h, 2 h, 4 h, 8 h, 12 h, 24 h) after administration of QSI-5 and SDM. Our results showed that QSI-5 was rapidly absorbed (0.5-h posttreatment [HPT]), reached the peak concentration at 2 HPT with a short half-life, and was excreted by 24 HPT (Fig. 6A), while the absorption of SDM was slower (2 HPT) and peak concentration was reached at 24 HPT (Fig. 6B). Additionally, the maximum concentration of QSI-5 in plasma (*Cmax*) values (0.76 ng/ML) was observed at 2 HPT, whereas the *C_max_* of SDM (933.6 ng/ML) was observed at 24 HPT.

Further, the safety of QSI-5 was demonstrated by measuring the level of drug residue in the muscle, liver, and kidney. Tissue samples (five chickens/group) were collected at 2-day post last treatment (DPLT), 5 DPLT, and 35 DPLT (slaughter age). Interestingly, no QSI-5 residue (0 ppm) in the muscle, liver, and kidney of treated chickens was detected at all time points. On the contrary, SDM residue was 0.17 ppm, 0.15 ppm, and 0.99 ppm at 2 DPLT and 0.0 ppm, 0.04 ppm, and 0.09 pp at 5 DPLT in muscle, liver and kidney, respectively. At 35 DPLT, no detectable residues of SDM were observed in any tissues (Table 2).

**TABLE 2:** Drug accumulation in the kidney, liver, and muscle in QSI-5- and sulfadimethoxine-treated groups^*a*^

Treatment group	Time point	Muscle (ppm± SD)	Liver (ppm± SD)	Kidney (ppm± SD)
QSI-5	2 DPLT	0.0 ± 0.0	0.0 ± 0.0	0.0 ± 0.0
	5 DPLT	0.0 ± 0.0	0.0 ± 0.0	0.0 ± 0.0
	35 DPLT (slaughter age)	0.0 ± 0.0	0.0 ± 0.0	0.0 ± 0.0
Sulfadimethoxine	2 DPLT	0.17 ± 0.17	0.15 ± 0.04	0.99 ± 0.15
	5 DPLT	0.02 ± 0.02	0.04 ± 0.02	0.09 ± 0.05
	35 DPLT (slaughter age)	0.0 ± 0.0	0.0 ± 0.0	0.0 ± 0.0

a DPLT, day post last treatment.

## DISCUSSION

Previously, we identified novel quorum sensing inhibitors (QSI) that did not affect the growth of APEC, but impacted the QS- regulated processes and showed promising efficacy against APEC infections *in vitro* (17). Using QSIs is an important approach to attenuate APEC pathogenicity and reduce the probability of development of resistant APEC strains. Interestingly, out of the seven QSIs tested in our study, two QSIs (QSI-5 and QSI-10) showed promising anti-APEC efficacy in chickens by (i) reducing the mortality up to 100% (Fig. 2A); (ii) reducing APEC load in the internal organs up to 6 logs (Fig. 2B); (iii) minimizing the pathological lesion severity in the internal organs up to 100% compared with the PC group (Fig. 2C); and (iv) maintaining body weight gain consistent with the NC group (Fig. 2D). Both QSI-5 and QSI-10 belong to the same chemical class with phenyl piperazinyl functional groups (17). Previously, amoxicillin and surfactin combination has been reported to reduce the mortality and bacterial loads in the liver of 1-day-old broiler chickens infected subcutaneously (s/c) with APEC and vaccinated with Marek’s disease vaccine (18), while oxytetracycline, trimethoprim-sulfadimethoxine, and enrofloxacin have been reported to reduce mortality and pathological lesions severity in 1-day-old chickens infected with APEC via airsac and vaccinated using infectious bronchitis vaccine (19). Similarly, ciprofloxacin has been reported to reduce mortality and APEC load in the liver of 1-day-old broilers infected orally with APEC O157 (20). Our study suggests that QSIs can be promising lead compounds to control the mortality and carcass condemnation due to APEC infection in poultry flocks without affecting the production performance. The challenge model used in this study was the s/c route using 10^7^ CFU/bird of APEC, which is considered as an acute infection model resulting in rapid progression of disease and high mortality (21). However, in the field conditions, chickens are exposed to much lower APEC (10^2^ to 10^3^) doses via oral or aerosol routes and the APEC infection develops slowly (21, 22). Therefore, these QSIs (QSI-5 and QSI-10) might be more effective in controlling APEC infection in chickens, if they are applied in field simulated settings using the oral (natural) route of infection, or when they are modified to improve oral bioavailability in chickens.

Oral administration of antimicrobials has been shown to affect the gut microbial community and immune responses (23, 24). Gut microbiota protects the host from pathogenic bacteria colonization and serve as source of amino acids, vitamins, enzymes, and short chain fatty acids to the host (25, 26). The misuse of antibiotics may affect the abundance of microbial species, microbial diversity, leading to increased susceptibility to pathogenic bacteria (27). This alteration might result in inflammation of the gut mucosal epithelium and subsequently mucosal colonization by pathogens (28). In this study, QSIs did not cause an alteration in the diversity of the gut microbial community. Treatment of chickens with QSI-5 and QSI-8 increased the Firmicutes (91.4% to 95%); whereas QSI-10 increased the Proteobacteria (8.6%-13.7%) compared with the NC group (Fig. 3A). Similar results were previously obtained in the gut microbiota of chickens after administration of antibiotics, small molecules, peptides, and probiotics (29–35). Previous reports have shown that increased Firmicutes abundance in the gut positively correlated with feed efficiency and chicken’s performance (36, 37). Therefore, we suggest that increase in the chicken’s body weight in QSI-5-, QSI-8-, and QSI-10-treated groups (Fig. 2D) might be due to the high abundance of Firmicutes population in the cecum. Notably, QSI-8 and QSI-10 significantly increased the abundance of *Butyricicoccus* (>4 folds) and *Lactobacillus* (>30 folds) genera in the gut (*P* < 0.05), respectively (Fig. 3B). *Lactobacillus* plays a role in enhancing innate and adaptive immunity, attenuating the inflammatory processes, and inhibiting pathogens growth (38, 39). Further, QSI-8 increased butyrate-producing bacteria, *Butyricicoccus* which plays a role in cell permeability and intestinal barrier functions (40). Previously, *B. pullicaecorum* has been reported to reduce Salmonella, Campylobacter, and Clostridium perfringens infections in chickens (41, 42). In a similar study, small molecules treatment increased the abundance of *Butyricicoccus* in the gut of chickens infected with Salmonella (30). We suggest that the anti-APEC activity of QSI-8 and QSI-10 may be enhanced by their growth-promoting effect on *Butyricicoccus* and *Lactobacillus*, respectively.

Interestingly, QSI-5 reduced levels of 5-methylthioadenosine (MTA) up to 7 folds (Table 1). The Methylthioadenosine/S-adenosylhomocysteine nucleosidase (MTA/SAH or MTAN) is involved in the AI-2 methylation cycle. MTAN is involved in 5′-methylthioadenosine recycling to S-adenosylmethionine (43) and inhibition of MTA/SAH resulted in suppression of AI-2 and subsequently reduced biofilm formation and bacterial virulence (44). The MTA/SAH or MTAN nucleosidase inhibitors were reported to be effective against Borrelia burgdorferi (45), Helicobacter pylori (43), Mycobacterium tuberculosis (46), and E. coli (47). Therefore, we suggest that the anti-quorum sensing efficacy of QSI-5 might be attributed to its inhibitory effect on the 5′-methylthioadenosine; however, additional studies are necessary to confirm this hypothesis and define the mechanisms of action of QSI-5. Additionally, the treatment of chickens with QSI-8 significantly increased the abundance of 9,10-DiHODE, a member of the class of linoleic acids, by 3.6 folds (Table 1). Linoleic acids are involved in lipid transport, lipid metabolism, and fatty acid metabolism. Several strains of gut bacteria have the ability to metabolize linoleic acids including *Lactobacilli*, *Lactococcus*, *Propionibacteria*, *Bifidobacteria*, *Faecalibacteria*, *Eubacteria*, *Anaerostipes*, *Roseburia*, *Clostridium*, and *Butyrivibrio* (48). Previously, it has been reported that Gamma-linolenic acid has anti-inflammatory effects on broiler chickens’ gut (49). Further, *Lactobacillus* strains producing conjugated linolenic acid have efficacy against enterohemorrhagic E. coli (50). The deficiency of linoleic acid in the diet of young chickens leads to reduced growth rate, enlarged liver, and reduced resistance to respiratory infections, while the deficiency of linoleic acid in laying hens results in reduced egg production, laying of small sized eggs, and reduced fertility and hatchability (51). We suggest that the increased level of linoleic acids in chicken’s serum might be due to the high abundance of butyrate-producing bacteria such as *Butyricicoccus* and *Lactobacillus* in chicken’s gut that is caused by QSI-8 (Fig. 3B).

In our study, QSI-5 possessed higher anti-APEC efficacy compared with SDM when administered at 1 mg/L in drinking water in a field simulated condition. QSI-5 demonstrated a 72.2% reduction in mortality, up to 2.8 logs reduction in APEC load, and up to 88.4% reduction in lesion severity (Fig. 5). The QSI-5 showed better efficacy even at a dose lower than SDM, an antibiotic used to treat APEC infections in poultry (19, 52). Further, QSI-5 was absorbed quickly into the blood circulation (at 0.5 HPT) and reached the peak concentration after 2 HPT. QSI-5 also showed good aqueous solubility, based on the observations during dosing in drinking water. These properties indicate that QSI-5 might have high bioavailability compared with SDM (53). No accumulated QSI-5 residues were detected in the edible tissues of chickens (Table 2), suggesting safety of the treated chickens for human consumption (54). Notably, the dose of QSI-5 (1 mg/L) is very low (up to 4,500 times) compared with the doses of antibiotics that are commonly used in the poultry industry to treat APEC such as SDM (495 mg/L), chlortetracycline (4.5 g/L), ampicillin (1.65 g/L), and sulfaquinoxaline (200 mg/ L) (55). This has a significant importance in terms of treatment costs and accumulation of drug residues in chickens tissues which is crucial for the safety of food for human consumption (56). Interestingly, increasing the dose of QSI-5 did not result in better anti-APEC efficacy in chickens. Furthermore, resistance to QSI-5 is less likely to occur as it does not affect the growth of APEC. Therefore, QSI-5 can be developed as an alternative to the current treatments for APEC infections in poultry in the field. Though, previously we have shown that these QSIs including QSI-5 are effective against multiple APEC serotypes *in vitro* (17), further studies are needed to demonstrate the effect of QSI-5 in chickens infected with other APEC serotypes that are implicated in colibacillosis.

In summary, our studies showed that QSI-5 is a promising novel anti-APEC therapeutic. QSI-5 showed the best anti-APEC efficacy among other tested QSIs in chickens with an optimal dose of 1 mg/L. Further, QSI-5 possessed higher anti-APEC efficacy compared with SDM in infected chickens with no impact the BWG, FCR, and cecal microbiota of the treated chickens. Further, there were no detectable residues in muscle, liver, and kidney. Our future studies will focus on testing QSI-5 in APEC-infected chickens using the natural route of infection in a field simulated conditions, improving the QSI-5 efficacy using medicinal chemistry, and elucidating the mechanisms of action of QSI-5. Furthermore, APEC shares genetic similarity to human ExPECs; therefore, our findings will have implications for developing novel antibacterials against human ExPEC infections.

## MATERIALS AND METHODS

### Ethics statement.

All the experimental procedures were carried out in accordance with approved Ohio State University Institutional Animal Care and Use Committee (IACUC) guidelines under protocol number 2010A00000149. The experiments were conducted according to approved husbandry practices.

### Bacterial inoculums and culture conditions.

APEC O78 (GenBank accession no. CP004009) (Tim Johnson, University of Minnesota, Saint Paul, MN, USA) (57). Rifampicin resistant APEC O78 (Rif^R^) was isolated on Luria-Bertani (LB) agar (Sigma-Aldrich, Inc. MO, USA)containing 50 μg/mL of rifampicin (EMD Millipore, USA) (58). For the preparation of bacterial inoculums, APEC O78 (Rif^R^) was grown overnight in LB media containing 50 μg/mL of rifampicin at 37°C with shaking at 200 rpm. The bacteria were then diluted 1:100 in fresh LB broth and was incubated with shaking at 200 rpm at 37°C for 3 h. Logarithmic phase grown culture of Rif^R^ APEC O78 (OD_600_ ~ 0.5) was washed twice with PBS and adjusted to the required concentration (OD_600_ = 0.1).

### Efficacy of QSIs in APEC-infected chickens.

The chicken experiment was carried out using 1-day-old broiler chickens (Mayer Hatchery, OH, USA). Feed and water were given *ad libitum*. Chickens (*n* = 6/group) were administered with the QSIs (QSI-1, QSI-2, QSI-5 − 8, QSI-10, ChemBridge, San Diego, CA); previously designated as C1, C2, C5, C6, C7, C8, C10 (17) using oral gavage. These compounds were selected based on their high efficacy in *in vitro* studies and the doses correspond to 30X of the initial *in vitro* screening concentration (17). The chemical structures of the QSIs used in this study are shown in Fig. 1. The QSIs were suspended in dimethyl sulfoxide (DMSO; used as a vehicle) and administered once daily for 5 successive days (starting from day 4 to day 8). The first dose was administered 1 day before the challenge, followed by the second dose on the challenge day (2 h before challenge), followed by three additional doses on subsequent days. Feeders were removed 1 h prior to the treatment and replaced 1 h after the treatment. The dose used for each QSI is shown in Table 3. Chickens were challenged subcutaneously (s/c) on day 5 with Rif^R^ APEC O78 (1 × 10^7^ CFU/bird) in PBS using syringe (27 gauge, 0.5 in.). The challenge dose, which reduced mortality by 50%, was chosen based on preliminary experiments conducted with different infection routes (subcutaneous s/c, intra-tracheal and intra-airsacs) and different doses (10^6^, 10^7^, and 10^8^ CFU/chicken). Chicken group infected s/c with 10^7^ CFU/bird possessed clear APEC colonization in liver, heart, lung, and kidney. Therefore, we selected this challenge dose and route for evaluation of QSIs in chickens (34). The positive (PC; infected with APEC O78 and DMSO treated) and negative (NC; non-infected and non-treated) control groups were included.

**TABLE 3:** Treatment groups and the dose of each AI-2 inhibitor

Groups	SM dose (μg/bird)	APEC O78
QSI-1	116.4	Yes
QSI-2	128	Yes
QSI-5	92.6	Yes
QSI-6	107.2	Yes
QSI-7	121.89	Yes
QSI-8	100.32	Yes
QSI-10	122.28	Yes
Positive control (PC)	DMSO	Yes
Negative control NC)	None	No

Chickens were monitored for clinical signs for 8 DPI. Any chickens moribund during this period were humanely euthanized and necropsied to determine bacterial load in the internal organs (liver, heart, lung, and kidney). Internal organs were aseptically collected, weighed and suspended in 1× PBS, the amount of PBS added was adjusted based on the organ size to make a suspension, the organ size and amount of PBS added were included in the final calculation of the CFU. The samples were homogenized, serially diluted 10-fold, plated on MacConkey agar (Remel, CA, USA) containing 50 μg/mL rifampicin, and incubated at 37°C for 24 h to determine the CFU/g. Pathological lesions severity in internal organs (pericarditis, perihepatitis, airsacculitis, and lung congestion) was scored as described previously (21, 59). At 8 DPI, the remaining chickens were humanely euthanized and necropsied, lesions were scored, and the APEC load was quantified in internal organs as described above.

### Quantification of serum metabolites.

In order to determine the effect of the QSI treatment on the metabolites present in chicken’s serum, untargeted metabolomic profiling was carried out using LC-MS as described before (34, 60). Blood was collected from the treated chicken groups that showed efficacy against APEC (QSI-5, QSI-8, and QSI-10-treated groups) and control groups (PC and NC), serum was separated and stored at −80°C for LC-MS analysis. Serum protein was precipitated by mixing cold methanol at −20°C for 30 min. The protein was then removed by centrifugation at 13,000 × *g* for 30 min. Five μL of the supernatant was transferred to glass vials for LC-MS analysis (2 to 3 runs for each sample). For quality control, equal portions of serum samples from QSIs-treated and control groups were pooled and run for LC-MS analysis every eight sample runs. Samples were run on a Thermo Orbitrap LTQ XL in positive mode analysis with high-performance liquid chromatography (HPLC) separation in a Poroshell 120 SB-C18 (2 × 100 mm in diameter, 2.7 μm particle sizes) columns using Thermo Fisher Scientific RLCS Ultimate 3000 LC system. The data analysis was performed in two separate groups (NC, QSI-5, PC) and (NC, QSI-8, QSI-10, PC). The metabolites were analyzed at the Campus Chemical Instrumentation Center, Mass Spectrometry and Proteomics Facility (CCIC, MS&PF), The Ohio State University (https://www.ccic.osu.edu/MSP). Progenesis QI (http://www.nonlinear.com/progenesis/qi/) and XCMS Online (https://xcmsonline.scripps.edu/) were used to identify the metabolites, retention time correction, feature detection, alignment, annotation, statistical analysis, and data visualization. The samples were aligned with a score of ≥ 88% and database matching was performed using the Human Metabolome Database, selecting for adducts M+H, M+Na, M+K, and M + 2H and <10 ppm mass error. Human metabolome database was used since no chicken metabolome database is available.

### Effect of the QSIs on the gut microbiota of chickens.

To determine the effect of the QSIs on the gut microbiota, metagenomic analysis targeting 16S rRNA was conducted as described previously (32). Genomic DNA extracted only from the cecum of the treated groups that possessed high efficacy against APEC (QSI-5, QSI-8, and QSI-10) and control groups (PC and NC) were analyzed. Quantitative Insights Into Microbial Ecology (QIIME 2) bioinformatics platform (61) was used for metagenomic analysis. DADA2 was used to create the feature table and make additional sequence filtering (62). The SILVA classifier was used for the taxonomy analysis, and the align-to-tree-mafft-fast tree pipeline was used for phylogenetic diversity analysis. The core-metrics-phylogenetic pipeline was used to analyze the alpha (Shannon’s diversity) and the beta diversity (Bray-Curtis distance).

### Optimization of the therapeutic dose of QSI-5 in drinking water.

QSI-5 showed the best activity against APEC when administered to chickens orally in the pilot study; therefore, the dose of QSI-5 was optimized for delivery in drinking water. One-day-old broiler chickens (*n* = 10/group) (Case Farms Ohio Hatchery, Strasburg, OH, USA) were used in this experiment. QSI-5 was synthesized in-house in the Fuchs laboratory (College of Pharmacy, OSU) via reductive amination reaction of commercially available 3-phenylpropionaldehyde with 1-(4-methylbenzyl) piperazine in the presence of acetic acid and sodium triacetoxyborohydride (Fig. S4). QSI-5 was administered daily in drinking water containing 0.05% DMSO at doses of 1 mg/L, 5 mg/L, 10 mg/L, and 20 mg/L for seven consecutive days starting from day 4 to day 10 of age. The amount of drinking water given daily was calculated based on the age of chickens (http://www.poultryhub.org/nutrition/nutrient-requirements/water-consumption-rates-for-chickens/). Chickens were infected with Rif^r^ APEC O78 (5 × 10^6^ CFU/chicken) on day 5 as described above. The PC (0.05% DMSO treated and infected) and NC (non-treated and non-infected) control groups were included. The clinical signs and the daily mortality were recorded for 8 DPI. Dead chickens were necropsied, and APEC load was quantified in the internal organs as mentioned above. At 8 DPI, the remaining chickens were necropsied, lesions were scored, and the APEC load was quantified in internal organs as described above.

### Comparative efficacy of QSI-5 and SDM in field simulated conditions.

To compare the efficacy of QSI-5 with an antibiotic SDM currently used in the field, chickens (*n* = 70) were raised on built-up floor litter in a field-simulated conditions. One-day-old broiler chickens (Case Farms Ohio Hatchery, OH, USA) were used for conducting the experiment. The optimized dose of QSI-5 (1 mg/L) and the therapeutic dose of SDM (495.323 mg/L) (0.05%) were given in drinking water daily for 7 days (starting from day 5 to day 11 of age). The volume of drinking water needed daily was determined as described above. On day 6, the chickens were infected s/c with Rif^r^ APEC O78 (5 × 10^6^ CFU/chicken, s/c). PC (0.05% DMSO treated and infected) and NC (non-infected and non-treated) control groups were included. The mortality was recorded daily until the end of the experiment (42 days of age: slaughter age). At 8 DPI, half of the chickens from each group were necropsied, lesions were scored, and the APEC load was quantified in the internal organs as described above. The other half of the chickens were raised until day 42 to determine the impact of treatment on the body weight gain and feed intake. To calculate the FCR, body weight was measured once every week and feed intake was recorded every day. On day 42, the remaining chickens were euthanized, body weight was measured and 10 chickens from each group were randomly selected and necropsied, lesions and APEC load were assessed in the internal organs.

### PK profile of QSI-5 and SDM.

The amount of QSI-5 and SDM in chicken plasma was measured using LC-MS. The optimized dose of QSI-5 (1 mg/L) and the therapeutic dose of SDM (495.323 mg/L) (0.05%) were administered orally as a single dose. Blood was collected individually from five chickens per group at different time points (0 h, 0.5 h, 1 h, 2 h, 4 h, 8 h, 12 h, 24 h) after administration of QSI-5 and SDM in vacutainer EDTA (10.8 mg) tubes (Becton, Dickinson, NJ, USA), blood was placed on ice for 1 h to clot, plasma was separated by centrifuging at 2,000 × *g* for 10 min at 4°C and stored at −80°C °C for LC-MS analysis. Plasma protein was precipitated by adding cold methanol and 10 μL (1 μg/mL) of an internal standard (IS) heavy-labeled phenylalanine (dissolved in 0.1% formic acid) was added to each 100 μL aliquot. The mixture was then incubated at −20°C for 30 min and centrifuged at 1300 × *g* for 25 min at 2°C. Sixty μL of the supernatant was pipetted into LC vials. For standard calibration, solutions of 0.0, 0.001, 0.005, 0.01, 0.05, 0.1, 0.5, 1, and 5 μg/mL of QSI-5 and SDM were prepared and 20 μL of each standard solution was added to 80 μL of cold methanol and IS as mentioned above. Calibration standard curves for QSI-5 and SDM are shown in Fig. S5A and S5B. All samples were run by injecting 5 μL on an Agilent Poroshell 120 SB-C18 (2 × 100 mm in diameter, 2.7 μm particle sizes). The LC system (Thermo Fisher Scientific UltiMate 3000 HPLC) was used with solvent A (10 mM ammonium formate and 0.1% formic acid) and solvent B (methanol with a flow rate of 200 μL/min). The gradient was set to 2% B at 2 min, increased from 2% to 20% at 5 min, 40% at 7.5 min, and reached 90% after 9 min. The gradient was sustained at 90% B for 11 min and then decreased to 2% at 12 min and was held for equilibration until the run ended after 15 min. The mass spectrometer (Thermo Fisher Scientific Quantiva Triple Quadrupole) was set to multiple reactions monitoring mode (SRM) with a heated electrospray ionization source (ESI) in positive mode at 3.5 kV. Drug targets were monitored for QSI-5 at transitions 309.26→105.1 *m/z* and 309.26→203.15 *m/z* at 28 and 20 V collision energy, respectively, and for SDM at transitions 311.11→156.11 *m/z* and 311.11→245.07 *m/z* at 21 and 18 V CE. The analysis of plasma samples was performed in Campus Chemical Instrumentation Center, Mass Spectrometry and Proteomics Facility (CCIC, MS&P), The Ohio State University (https://live-ccic.pantheonsite.io/MSP).

### QSI-5 and SDM residue quantification in muscle, kidney, and liver.

The safety of QSI-5 and SDM was assessed by measuring the level of drug residue in the muscle, kidney, and liver using LC-MS (CCIC, MS&P Facility, OSU) as described before (34). As described above, chickens were administered with the optimized dose of QSI-5 (1 mg/L) and the therapeutic dose of SDM (495.323 mg/L) (0.05%) in drinking water daily for 7 days (starting from day 5 to day 11 of age). Tissue samples were collected individually from chickens treated with QSI-5 and SDM (five chickens per group) from experimental set up as described for floor trial at 2 DPLT, 5 DPLT, and 35 DPLT (slaughter age). All samples were weighed and extracted at a 400 mg/mL ratio of tissue in the extraction solution (50:50 H_2_O: ACN). Samples were homogenized using a probe sonicator 20 times and centrifuged at 13,000 × *g* for 30 min. Sixty μL of the supernatant was transferred into glass vials and dried in a SpeedVac for 1.5 h, and then resuspended in 120 μL of 25:25:50 H_2_O:ACN:MeOH. Standard calibration, LC system, mass spectrometer, and drug target monitoring were performed as described above. Calibration standard curves for measuring the accumulation of QSI-5 and SDM in the kidney, liver, and muscle are shown in Fig. S6A and S6B.

### Statistical analysis.

Statistical analyses were conducted using ANOVA and the Tukey test in the GraphPad Prism 5 software (GraphPad, Inc., CA, USA). Differences in lesion scores, APEC load, BWG, and FCR between treatment and control groups were analyzed using the Mann-Whitney U test and the Kruskal-Wallis test. Differences in the OTU relative abundance between the treated and control groups were calculated using the Mann-Whitney U test. The alpha diversity was assessed using permutational multivariate analysis of variance (PERMANOVA) and the Kruskal-Wallis test. Statistically significant differences between means were determined using A *P*-value < 0.05. The statistical analysis of the metabolite intensity data was performed using JMP Pro14 and vegan package on Rstudio (SAS institute Inc., NC, USA). Distribution of the metabolites profile for each chicken was visualized using PCA. PERMANOVA was used to determine whether significant spatial distribution was observed between the chicken groups. Wilcoxon rank-sum test was used to identify intensity differences between QSI groups and the PC group for a designated metabolite (63). A threshold of *P*-value 0.001 was used to select the metabolites of interest.

### Data availability.

Data from the study are included in this article and in the supplementary files. Microbiome sequence data have been deposited in the BioProject database under accession number PRJNA766869.
