# Supplementary material for: Evaluation of Novel Quorum Sensing Inhibitors Targeting Auto-Inducer 2 (AI-2) for the Control of Avian Pathogenic Escherichia coli Infections in Chickens
Source: Microbiol Spectr. 2022 May 18;10(3):e00286-22. doi: 10.1128/spectrum.00286-22 (PMC9241644; doi:10.1128/spectrum.00286-22)
Supplement: SUPPLEMENTAL FILE 1 — Supplemental material. Download spectrum.00286-22-s001.pdf, PDF file, 0.7 MB [file spectrum.00286-22-s001.pdf]

Table S1. Mortality reduction, average APEC load reduction, and average lesion severity reduction in QSIs treated chickens

QSIs	Mortality rate % (Nr.)	Average APEC load (Log CFU/g of tissue)				Average lesion severity			
		Liver	Lung	Heart	Kidney	Liver	Lung	Heart	Airsac
**QSI-1**	50 (3/6)	5.30	5.92	5.63	6.28	2.17	0.50	3.00	1.83
**QSI-2**	33.3 (2/6)	4.20	5.15	4.87	5.07	1.00	0.17	1.67	0.92
**QSI-5**	0 (0/6)	0.72	0.75	0.58	0.67	0.33	0.17	0.33	0.17
**QSI-6**	66.6 (4/6)	4.62	5.17	4.87	5.35	0.83	0.50	1.50	1.17
**QSI-7**	66.6 (4/6)	5.95	6.10	6.13	6.13	1.33	0.17	1.33	1.00
**QSI-8**	33.3 (2/6)	3.48	4.60	3.60	3.65	0.50	0.17	0.50	0.50
**QSI-10**	16.6 (1/6)	2.50	3.08	2.50	2.47	0.00	0.00	0.33	0.17
**PC**	66.6 (4/6)	5.95	6.83	5.98	6.81	1.75	1.25	2.25	2.00

PC; positive control group (chickens infected with APEC and treated with 0.05% of DMSO). Lesions were scored as: air sacs, (1) slight opaque and/or thickened membranes ± slight amount of fibrin, (2) moderate opaque and/or thickened membranes ± moderate amount of fibrin, (3) severe opaque and/or thickened membranes ± severe amount of fibrin ± moderate to severe vascularization); Lung, (1) single small lesion, locally restricted (1/5 of the organ), (2) multiple, locally restricted small lesions and/or one bigger lesion (2/5 of the organ), (3) lesions cover about 1/2 of the organ, (4) lesions cover about 1/2 of the organ, (5) complete organ covered with lesions; Heart, (1) vascularization, opacity, (2) excessive or cloudy fluid in the pericardial cavity, (3) thickened pericardium, acute pericarditis; Liver, (1) decolorization and/or slight amounts of fibrinous exudates, (2) marked perihepatitis with high amounts of fibrinous exudates. The clinical signs and the daily mortality were recorded for 8 DPI.

Table S2. Mortality reduction, average APEC load reduction and average lesion severity reduction in QSI-5 treated chickens at 1 mg/L, 5 mg/L, 10 mg/L, 20 mg/L.

QSI-5 dose	Mortality rate % (Nr.)	Average APEC load (Log CFU/ g of tissue)				Average lesion severity			
		Liver	Lung	Heart	Kidney	Liver	Lung	Heart	Airsac
**1 mg/L**	37.5 (3/8)	5.33	5.68	5.71	5.06	1.25	0.33	1.63	1.38
**5 mg/L**	66.6 (6/9)	6.03	5.86	6.09	6.10	1.33	0.38	1.89	1.33
**10 mg/L**	44.4 (4/9)	5.84	6.37	7.32	6.20	2.11	0.78	2.44	2.33
**20 mg/L**	66.6 (6/9)	6.53	6.58	6.69	6.56	1.22	0.44	2.11	1.67
**PC**	88.9 (8/9)	8.46	7.93	8.37	7.84	2.11	1.11	2.78	3.11

PC; positive control group (chickens infected with APEC and treated with 0.05% of DMSO). Lesions were scored as: air sacs, (1) slight opaque and/or thickened membranes ± slight amount of fibrin, (2) moderate opaque and/or thickened membranes ± moderate amount of fibrin, (3) severe opaque and/or thickened membranes ± severe amount of fibrin ± moderate to severe vascularization); Lung, (1) single small lesion, locally restricted (1/5 of the organ), (2) multiple, locally restricted small lesions and/or one bigger lesion (2/5 of the organ), (3) lesions cover about 1/2 of the organ, (4) lesions cover about 1/2 of the organ, (5) complete organ covered with lesions; Heart, (1) vascularization, opacity, (2) excessive or cloudy fluid in the pericardial cavity, (3) thickened pericardium, acute pericarditis; Liver, (1) decolorization and/or slight amounts of fibrinous exudates, (2) marked perihepatitis with high amounts of fibrinous exudates. The clinical signs and the daily mortality were recorded for 8 DPI.

Table S3. Mortality reduction, average APEC load reduction and average lesion severity reduction in QSI-5 treated chickens compared to sulfadimethoxine (SDM).

QSI	Mortality rate% (Nr.)	Average APEC load (Log CFU/ g of tissue)				Average lesion severity			
		Liver	Lung	Heart	Kidney	Liver	Lung	Heart	Airsac
**QSI-5**	12.7 (8/63)	4.95	4.93	5.46	5.06	1.68	0.21	1.93	1.25
**SDM**	29.2 (19/65)	5.05	6.17	5.69	5.33	2.03	1.17	2.83	2.50
**PC**	45.6 (31/68)	7.24	7.68	7.92	7.73	2.52	1.85	3.58	3.36

PC; positive control group (chickens infected with APEC and treated with 0.05% of DMSO). Lesions were scored as: air sacs, (1) slight opaque and/or thickened membranes ± slight amount of fibrin, (2) moderate opaque and/or thickened membranes ± moderate amount of fibrin, (3) severe opaque and/or thickened membranes ± severe amount of fibrin ± moderate to severe vascularization); Lung, (1) single small lesion, locally restricted (1/5 of the organ), (2) multiple, locally restricted small lesions and/or one bigger lesion (2/5 of the organ), (3) lesions cover about 1/2 of the organ, (4) lesions cover about 1/2 of the organ, (5) complete organ covered with lesions; Heart, (1) vascularization, opacity, (2) excessive or cloudy fluid in the pericardial cavity, (3) thickened pericardium, acute pericarditis; Liver, (1) decolorization and/or slight amounts of fibrinous exudates, (2) marked perihepatitis with high amounts of fibrinous exudates. The mortality was recorded daily until the end of the experiment (42 days of age). Each group has a variable number of chickens as the weak chickens were removed from each group after setting up the experiment and before administering the first treatment.

Table S4: Weekly and overall BWG in treated and control groups.

Groups	Week 2	Week 3	Week 4	Week 5	Week 6	Overall BWG*
QSI-5	106.12	454.27	519.41	668.96	910.3	2659.1 (P=0.85)
SDM	105.1	307.3	454.4	610.3	931	2408.1 (P=0.46)
PC	121.9	383.5	458.2	496.2	853.0	2312.8 (P=0.09)
NC	157.8	489.9	490.1	610.8	752.7	2501.2

*Average body weight of week 1 (QSI-5: 54.27, SDM (sulfadimethoxine): 53.86, PC: 52.19, and NC: 50.27) was used to calculate overall BWG. The BWG in QSI-5, SDM, and PC groups was statistically non-significant (Student's t-test) compared to NC group.

Table S5: Weekly and overall FCR in treated and control groups.

Groups	Week 2	Week 3	Week 4	Week 5	Week 6	Overall FCR*
QSI-5	2.5	0.7	1.3	1.0	1.9	1.48 (P=0.94)
SDM	2.5	0.7	1.4	1.0	1.6	1.49 (P=0.64)
PC	2.2	0.9	1.9	1.3	1.4	1.51(P=0.43)
NC	2.0	0.7	1.4	1.1	2.0	1.45

* FCR was calculated by using formula: average overall feed intake/average BWG. FCR in QSI-5, sulfadimethoxine (SDM) and PC groups was statistically non-significant (Student's t-test) compared to NC group

Supplemental Figure Legends

Figure S1: The survival rate of the chickens after treatment with; 1) different quorum sensing inhibitors, 2) different doses of QSI-5 (the best quorum sensing inhibitor), and 3) QSI-5 and compare its efficacy with the currently used antibiotic (sulfadimethoxine; SDM) in field-simulated conditions.

Figure S2: Principal component analysis (PCA) of metabolites clustering in serum of; **(A)** QSI-5 (PC3=6.78%, PC5=3.5%), and **(B)** QSI-8, and QSI-10 (PC1=30.5%, PC7=3.97%) treated chickens compared to non-infected and non-treated (NC) and DMSO treated and infected (PC) chicken groups.

Figure S3: (A) Shannon's diversity index showing the microbial richness and evenness in QSIs treated groups compared to PC group. QSIs caused no significant differences in the richness and evenness of cecal microbiota compared to PC group. *$P<0.05$, Kruskal-Wallis test. (B) Impact of QSIs treatment on beta diversity of cecal microbiota of chickens. Beta diversity was evaluated using principal coordinates analysis of unweighted uniFrac values. There was no spatial separation observed in the cecal microbiota of the treated groups compared to PC and NC groups. Each dot represents cecum sample from each treatment groups.

Figure S4: Synthesis of QSI- 5 (1-(4-methylbenzyl)-4-(3-phenylpropyl)piperazine) using reductive amination. 1-(4-Methylbenzyl)piperazine (50 mg, 0.26 mmol) and 3-phenylpropionaldehyde (35 mL, 0.26 mmol) were dissolved in DMF (1.32 mL) at room temperature with stirring. Acetic acid (15 mL, 0.26 mmol) was added to the mixture, followed by sodium triacetoxyborohydride (111.5 mg, 0.526 mmol). The resulting solution was allowed to stir for an additional 2.5 hours and then quenched with H_2O. The mixture was extracted three times with EtOAc and the combined layers were dried over sodium sulfate and concentrated in vacuo. The crude mixture was then purified using silica gel column chromatography (95:5 – 90:10 gradient, dichloromethane:methanol) to provide 36 mg (0.12 mmol, 44%) of the desired product. ^{1}H NMR (400 MHz, MeOD) δ 7.27 – 7.10 (m, 9H), 3.47 (s, 2H), 2.61 (t, J = 7.6 Hz, 2H), 2.49 (s, 8H), 2.39 – 2.33 (m, 2H), 2.31 (s, 3H), 1.86 – 1.76 (m, 2H); ^{13}C NMR (101 MHz, MeOD) δ 141.76, 136.81, 133.63, 129.34, 128.51, 128.00, 127.96, 125.47, 62.21, 57.66, 52.41, 52.11, 33.27, 27.92, 19.72.

Figure S5: Calibration **s**tandard curves for determining the concentration of; A) QSI-5 and B) SDM in chicken's blood using LC-MS. For standard calibration, solutions of 0.0, 0.001, 0.005, 0.01, 0.05, 0.1, 0.5, 1, and 5 μg/mL of QSI-5 and SDM were used.

Figure S6: Calibration standard curves for determining the bioaccumulation of QSI-5 and SDM in chicken's; A) Kidney and B) liver and muscle using LC-MS.

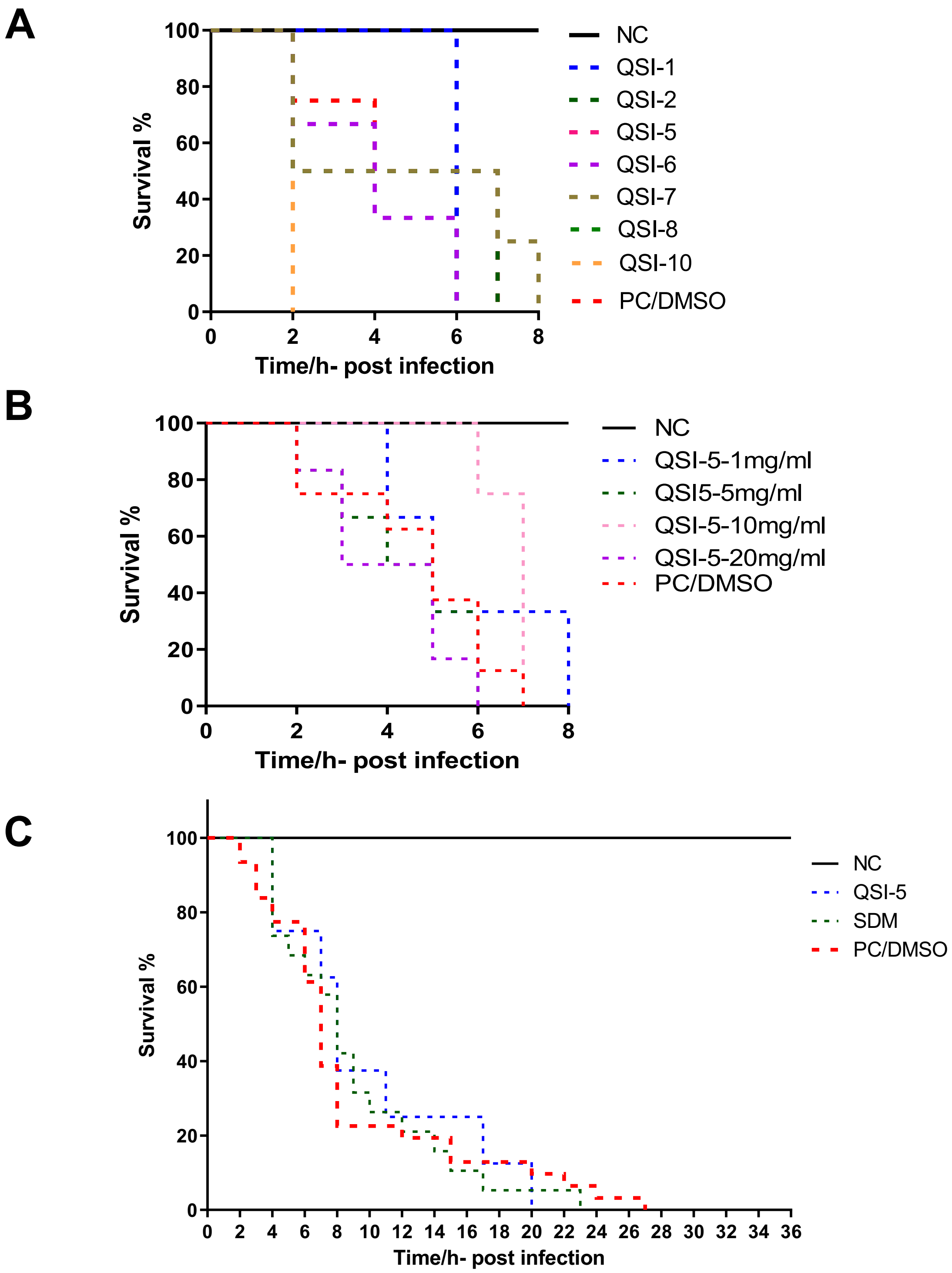
A
Survival %
100
80
60
40
20
0
0 2 4 6 8
Time/h- post infection
NC
QSI-1
QSI-2
QSI-5
QSI-6
QSI-7
QSI-8
QSI-10
PC/DMSO
B
Survival %
Time/h- post infection
NC
QSI-5-1mg/ml
QSI5-5mg/ml
QSI-5-10mg/ml
QSI-5-20mg/ml
PC/DMSO
C
Survival %
0 2 4 6 8 10 12 14 16 18 20 22 24 26 28 30 32 34 36
Time/h- post infection
NC
QSI-5
SDM
PC/DMSO

A

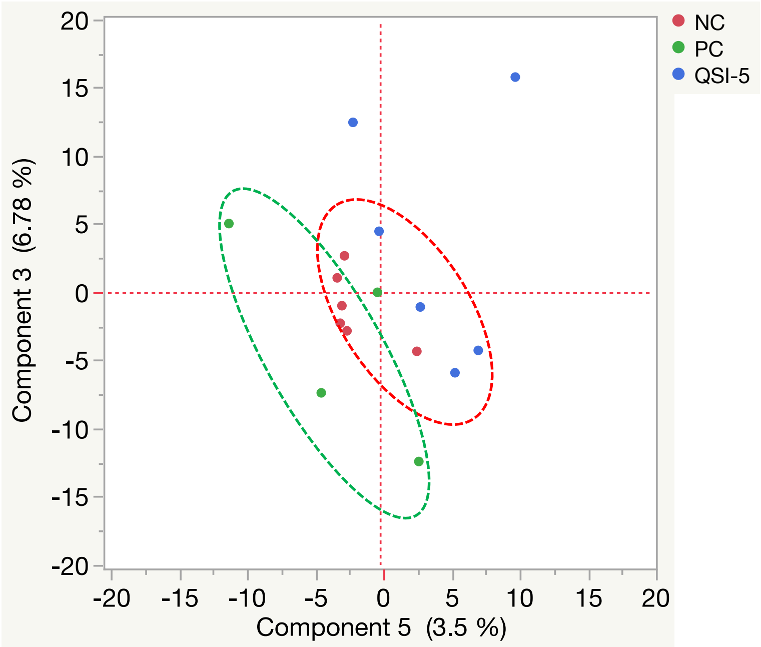

NC
PC
QSI-5
Component 3 (6.78 %)
Component 5 (3.5 %)
20
15
10
5
0
-5
-10
-15
-20

B

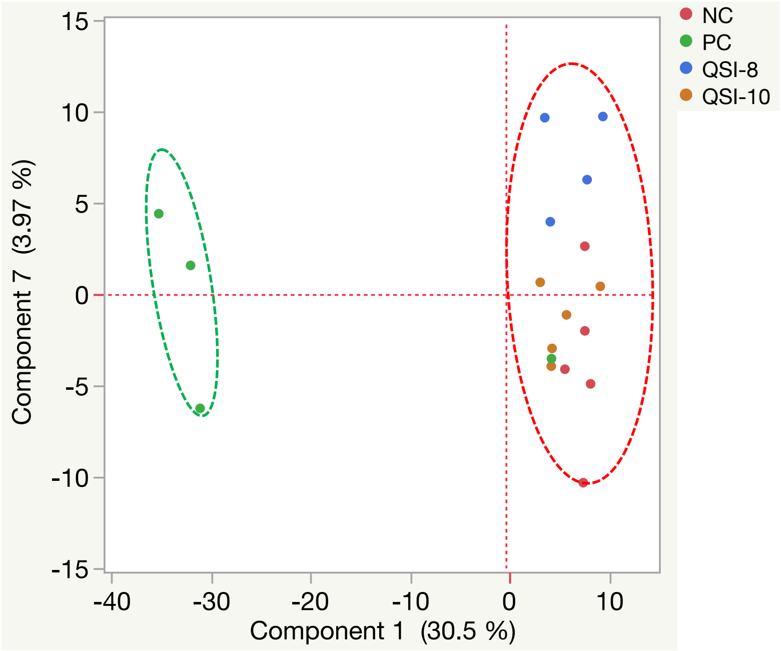

NC
PC
QSI-8
QSI-10
Component 7 (3.97 %)
Component 1 (30.5 %)
15
10
5
0
-5
-10
-15
-40
-30
-20
-10
0
10

A

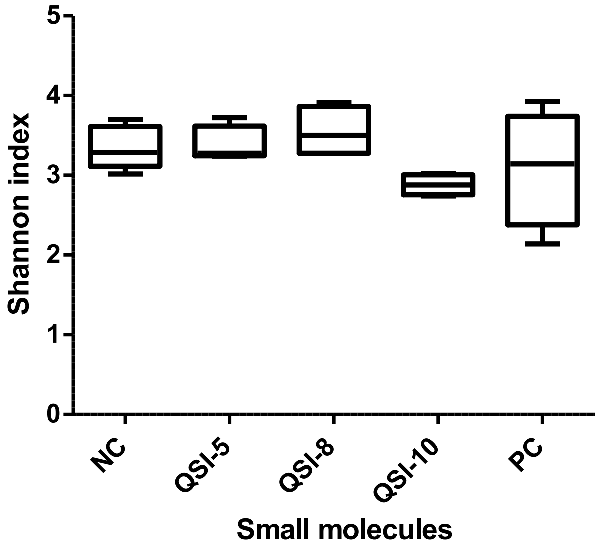

5
4
3
2
1
0
Shannon index
NC
QSI-5
QSI-8
QSI-10
PC
Small molecules

B

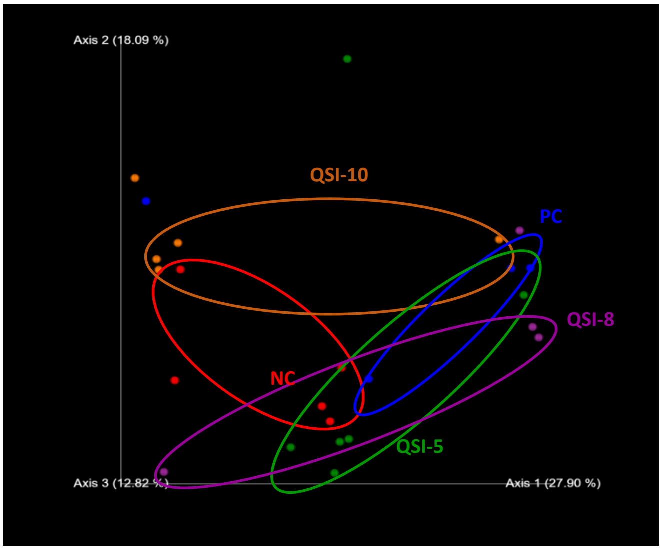

Axis 2 (18.09 %)
QSI-10
PC
QSI-8
NC
QSI-5
Axis 3 (12.82 %)
Axis 1 (27.90 %)

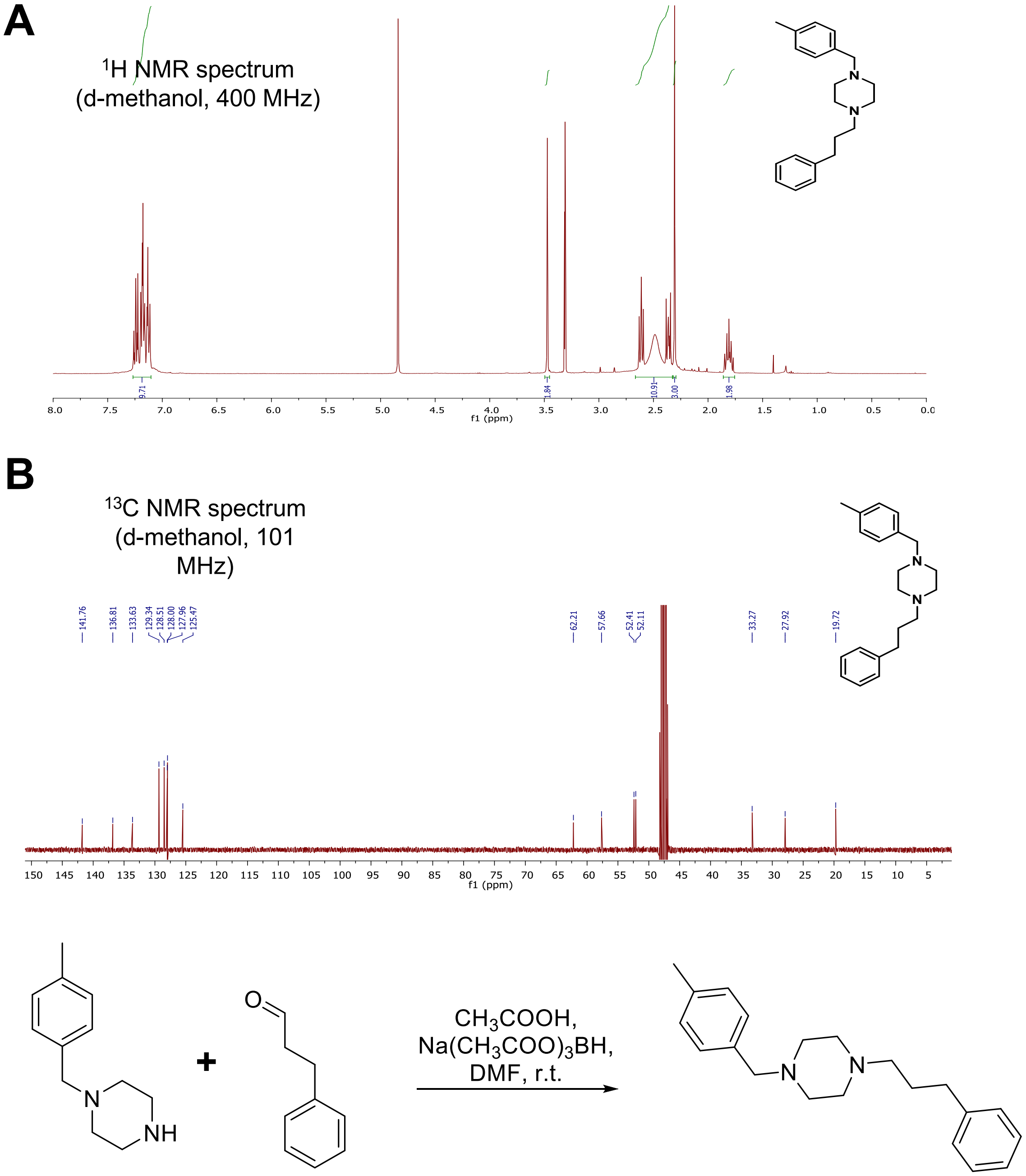
A
1H NMR spectrum
(d-methanol, 400 MHz)
9.71
1.84
10.91
3.00
1.98
8.0 7.5 7.0 6.5 6.0 5.5 5.0 4.5 4.0 3.5 3.0 2.5 2.0 1.5 1.0 0.5 0.0
f1 (ppm)
B
13C NMR spectrum
(d-methanol, 101
MHz)
141.76
136.81
133.63
129.34
128.51
128.00
127.96
125.47
62.21
57.66
52.41
52.11
33.27
27.92
19.72
150 145 140 135 130 125 120 115 110 105 100 95 90 85 80 75 70 65 60 55 50 45 40 35 30 25 20 15 10 5
f1 (ppm)
N
NH
O
CH3COOH,
Na(CH3COO)3BH,
DMF, r.t.
N
N

A

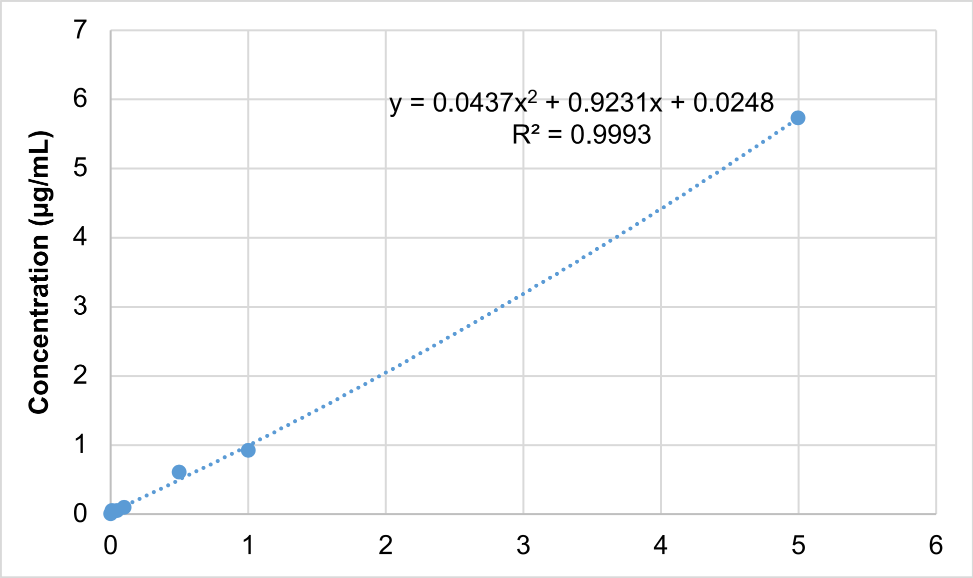

Concentration (µg/mL)
7
6
5
4
3
2
1
0
0
1
2
3
4
5
6
y = 0.0437x2 + 0.9231x + 0.0248
R² = 0.9993

B

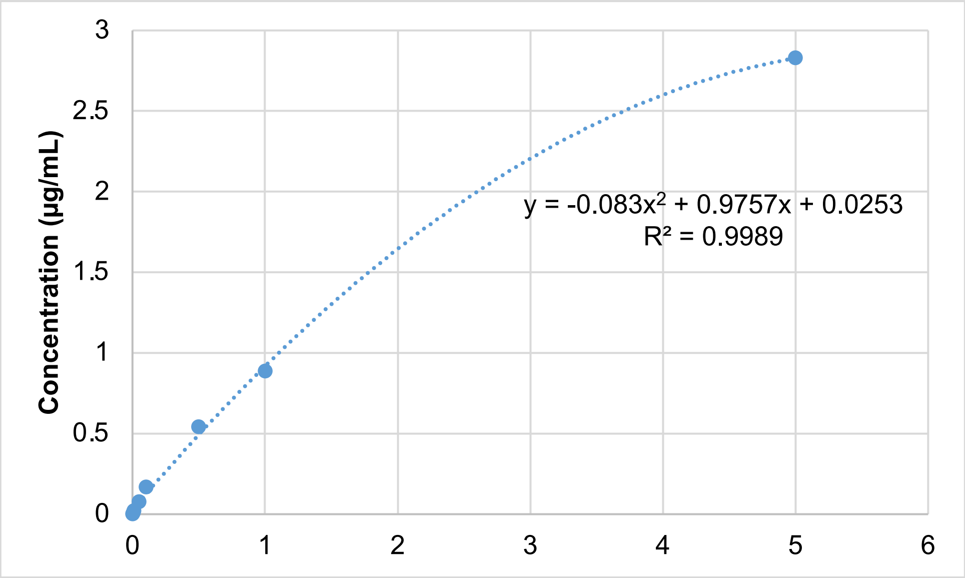

Concentration (µg/mL)
3
2.5
2
1.5
1
0.5
0
0
1
2
3
4
5
6
y = -0.083x2 + 0.9757x + 0.0253
R² = 0.9989

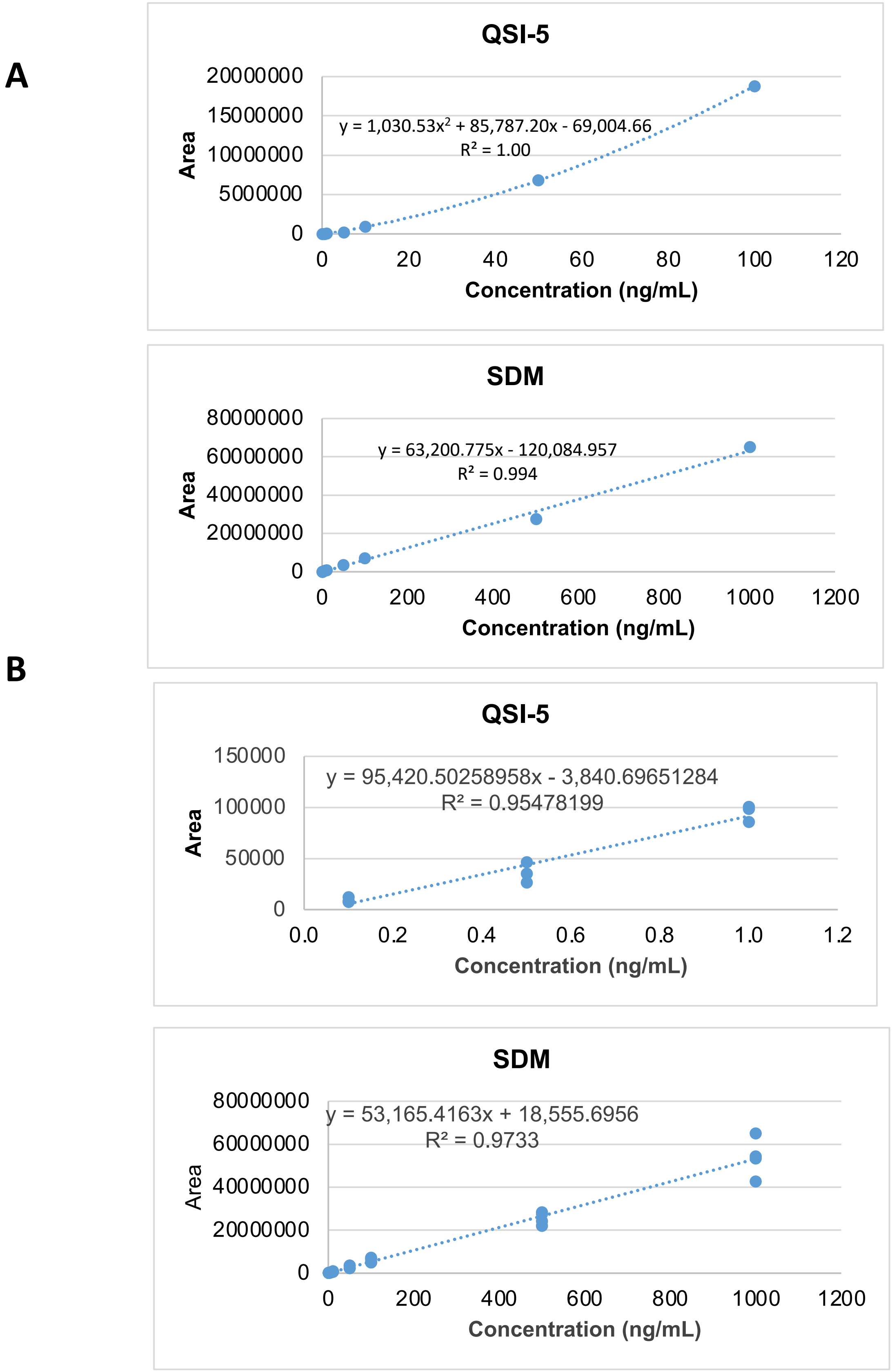
A
QSI-5
20000000
15000000
10000000
5000000
0
Area
y = 1,030.53x² + 85,787.20x - 69,004.66
R² = 1.00
0
20
40
60
80
100
120
Concentration (ng/mL)
SDM
80000000
60000000
40000000
20000000
0
Area
y = 63,200.775x - 120,084.957
R² = 0.994
0
200
400
600
800
1000
1200
Concentration (ng/mL)
B
QSI-5
150000
100000
50000
0
Area
y = 95,420.50258958x - 3,840.69651284
R² = 0.95478199
0.0
0.2
0.4
0.6
0.8
1.0
1.2
Concentration (ng/mL)
SDM
80000000
60000000
40000000
20000000
0
Area
y = 53,165.4163x + 18,555.6956
R² = 0.9733
0
200
400
600
800
1000
1200
Concentration (ng/mL)
